# DNA Methylation Mediated Control of Gene Expression Is Critical for Development of Crown Gall Tumors

**DOI:** 10.1371/journal.pgen.1003267

**Published:** 2013-02-07

**Authors:** Jochen Gohlke, Claus-Juergen Scholz, Susanne Kneitz, Dana Weber, Joerg Fuchs, Rainer Hedrich, Rosalia Deeken

**Affiliations:** 1 Julius-von-Sachs-Institute, Department of Molecular Plant Physiology and Biophysics, University of Wuerzburg, Wuerzburg, Germany; 2 IZKF Laboratory for Microarray Applications, University Hospital of Wuerzburg, Wuerzburg, Germany; 3 Physiological Chemistry I, Biocenter, University of Wuerzburg, Wuerzburg, Germany; 4 Leibniz Institute of Plant Genetics and Crop Plant Research (IPK), Gatersleben, Germany; Virginia Tech, United States of America

## Abstract

Crown gall tumors develop after integration of the T-DNA of virulent *Agrobacterium tumefaciens* strains into the plant genome. Expression of the T-DNA–encoded oncogenes triggers proliferation and differentiation of transformed plant cells. Crown gall development is known to be accompanied by global changes in transcription, metabolite levels, and physiological processes. High levels of abscisic acid (ABA) in crown galls regulate expression of drought stress responsive genes and mediate drought stress acclimation, which is essential for wild-type-like tumor growth. An impact of epigenetic processes such as DNA methylation on crown gall development has been suggested; however, it has not yet been investigated comprehensively. In this study, the methylation pattern of *Arabidopsis thaliana* crown galls was analyzed on a genome-wide scale as well as at the single gene level. Bisulfite sequencing analysis revealed that the oncogenes *Ipt, IaaH*, and *IaaM* were unmethylated in crown galls. Nevertheless, the oncogenes were susceptible to siRNA–mediated methylation, which inhibited their expression and subsequently crown gall growth. Genome arrays, hybridized with methylated DNA obtained by immunoprecipitation, revealed a globally hypermethylated crown gall genome, while promoters were rather hypomethylated. Mutants with reduced non-CG methylation developed larger tumors than the wild-type controls, indicating that hypermethylation inhibits plant tumor growth. The differential methylation pattern of crown galls and the stem tissue from which they originate correlated with transcriptional changes. Genes known to be transcriptionally inhibited by ABA and methylated in crown galls became promoter methylated upon treatment of *A. thaliana* with ABA. This suggests that the high ABA levels in crown galls may mediate DNA methylation and regulate expression of genes involved in drought stress protection. In summary, our studies provide evidence that epigenetic processes regulate gene expression, physiological processes, and the development of crown gall tumors.

## Author Summary

Until now, knowledge about the impact of DNA methylation on plant tumor development and physiology has been scant. Therefore, we studied the methylation pattern of *Arabidopsis thaliana* crown galls on a genome-wide and single-gene level. Crown gall tumor development requires expression of oncogenes, which are transferred on T-DNA of virulent *Agrobacterium tumefaciens* strains into the plant genome. We found that oncogene expression was associated with an unmethylated oncogene sequence although the promoters were susceptible to methylation. siRNA–mediated promoter methylation caused transcriptional silencing of oncogenes and prevented crown gall proliferation. Moreover, we observed that the genome-wide DNA methylation profile of crown gall tumors was significantly altered and influenced gene expression pattern as well as tumor development. Finally, we demonstrated that physiological processes important for wild-type-like crown gall growth, such as abscisic acid-dependent drought stress protection, are regulated by DNA methylation. From our data, we conclude that epigenetic processes control gene expression, development, and physiology of crown gall tumors.

## Introduction

The bacterial pathogen *A. tumefaciens* genetically engineers the host plant by transferring its T-DNA, a piece of DNA from the tumor-inducing (Ti) plasmid, into the plant genome. Expression of the T-DNA-encoded oncogenes *IaaH*, *IaaM*, and *Ipt* results in increased synthesis of both auxin and cytokinin [1], [2]. High concentrations of these phytohormones not only facilitate proliferation of transformed plant cells, but also differentiation of specialized cell types within the resulting crown gall tumor [3]. In addition to auxin and cytokinin, elevated levels of the phytohormones salicylic acid, ethylene and abscisic acid (ABA) have been observed in crown galls on *A. thaliana* stems [4]–[6]. In particular, ABA was shown to be important for drought stress acclimation to ensure wildtype-like crown gall growth [7]. Moreover, approximately 20% of protein coding genes are differentially transcribed in these plant tumors compared to tumor-free stem tissue [8]. The massive changes in gene expression, together with the cooperative action of phytohormones, fulfill distinct roles in differentiation, pathogen defense, metabolic changes, and physiological adaptations in crown galls [3], [7], [8]. In recent years, there has been increased interest in the role of epigenetic events in regulating biotic and abiotic stress responses in plants [9]. Environmental stresses have been shown to influence epigenetic processes, inducing the release of transcriptional silencing of transgenes and several endogenous *A. thaliana* gene loci.

Changes in the DNA methylation pattern have also been reported in cases where, similar to *A. thaliana* crown galls, foreign DNA is integrated into the mammalian genome prior to tumor formation. For example, mammalian tumors induced by adenovirus type 12 display extensive genome-wide hypermethylation [10]. These widespread differences in the methylation pattern during mammalian tumor formation indicate that they may be a common feature of neoplastic growth, possibly also during plant tumor development. Such an epigenetic contribution to crown gall formation was already suggested by Braun 50 years ago [11]. To date, only the integrated T-DNA has been examined with respect to DNA methylation. The T-DNA of different crown gall lines was shown to be frequently methylated. At least one T-DNA copy in each tumor genome remained unmethylated [12], [13], which allowed expression of oncogenes and thereby crown gall proliferation. T-DNA methylation can be induced by siRNAs which are produced by dicer activities on long dsRNA. Synthesis of the latter RNAs results from bidirectional or read-through transcription of rearranged or integrated T-DNAs. While siRNAs corresponding to T-DNA oncogenes accumulate in *A. tumefaciens*-infected plant tissue, synthesis of siRNA is specifically inhibited in developing tumors resulting in a potent antisilencing state [14].

In the *A. thaliana* genome, the highest levels of methylation are found in transposon-rich heterochromatic regions. This methylation pattern is in agreement with a primary function for methylation in transposon silencing. However, DNA methylation of protein coding genes also frequently occurs. Methylation is depleted at promoters and gene ends, indicating that it interferes with important regulatory functions in these gene segments [15]. Endoreduplication is also known to cause methylation changes as a result of increased ploidy levels in *A. thaliana* [16]. Furthermore, endoreduplication is a phenomenon known to occur in specialized cell types of animals and in different tissues of many plant species [17]. It extensively occurs in *A. thaliana*, especially if the levels of auxin and cytokinin are increased, such as in crown galls [18], [19].

Methylation in plants differs from that in mammals in its sequence context. In plants, cytosines are methylated in three different sequence contexts (CG, CHG and CHH, where H = A, C, T), whereas methylation at CG dinucleotides predominates in mammals [20]. DNA methylation in *A. thaliana* is established by DRM1 and DRM2 (DOMAINS-REARRANGED-METHYLASE) methyltransferases in all sequence contexts [21]. Methylation of specific genomic regions can be targeted by DRM proteins through interaction with ARGONAUTE4 (AGO4). Small RNAs of 21–24 nt are incorporated in AGO4 and guide DRM activity to the corresponding genomic sequences [22]. Both DRM methyltransferases and AGO4 are also essential for transgene silencing which is inducible by hairpin constructs complementary to a transgene promoter [23]. CG methylation is maintained during genome replication by the activity of MET1 (METHYLTRANSFERASE1; [24]), while the plant-specific DNA methyltransferase CMT3 (CHROMOMETHYLASE) primarily methylates cytosines in the CHG context [25]. Furthermore, subsets of genomic DNA methylation patterns are influenced by the activity of the demethylating enzymes ROS1 (REPRESSOR OF SILENCING), DME (DEMETER), DML2 (DEMETER-LIKE) and DML3 [26].

Our study provides the results of a genome-wide methylation analysis of stem-derived *A. thaliana* crown galls in comparison with mock-inoculated stem tissue. This analysis indicates that the crown gall tumor genome is globally hypermethylated, while promoter regions are hypomethylated. These changes in the DNA methylation pattern seem to exert an inhibitory influence on growth of crown gall tumors, since *A. thaliana* mutants with reduced DNA methylation, like *drm1/drm2/cmt3* (*ddc) and ago4,* developed significantly larger crown galls. The global differences in DNA methylation between the crown gall and the tumor-free stem genome were in agreement with the transcriptomic changes of protein coding genes. For example, genes involved in ABA-dependent drought stress protection were promoter methylated and transcriptionally silenced in crown gall tumors. *A. thaliana* seedlings treated with the stress phytohormone demonstrated ABA-dependent methylation of the promoters of these genes. Taken together, our studies provide evidence for a role of epigenetic processes in controlling gene expression, development and physiology in crown gall tumors.

## Results

### Methylation of the T-DNA–encoded oncogenes

**Figure 1:**
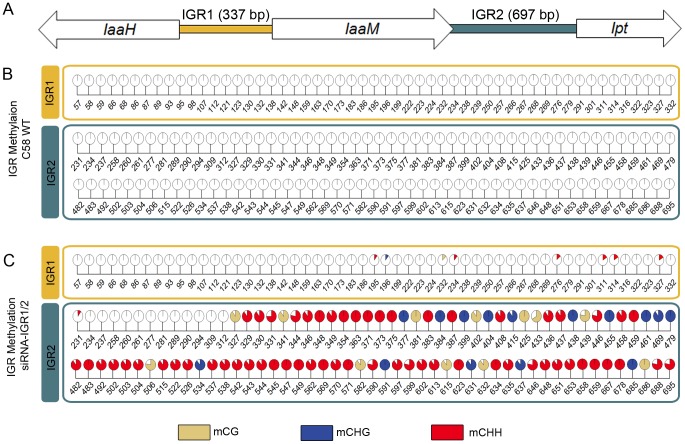
Intergenic regions (IGRs) of the T-DNA–encoded oncogenes become methylated upon an siRNA trigger. (A) IGRs upstream of the oncogenes *IaaH*, *IaaM* (IGR1) and *Ipt* (IGR2) from the T-DNA of pTiC58 were analyzed by applying bisulte sequencing (model not drawn to scale). Coding sequences for *IaaH*, *IaaM* and *Ipt* are depicted as arrows, colored bars illustrate the two IGRs. (B) Detailed map of all methylated cytosines within IGR1 and IGR2 in the crown gall tumor induced by the wildtype *A. tumefaciens* strain C58 (C58 WT) and (C) of plant material inoculated with the same strain C58, but in addition harboring a binary vector with a hairpin construct directed against both IGRs (siRNA-IGR1/2). Percentages of methylation at each position are visualized by pie charts filled with different colors for the three methylation motifs (mCG brown, mCHG blue, mCHH red). Numbers below pie charts indicate nucleotide positions from the start of the analyzed region. Cytosines outside of the displayed regions were unmethylated. Percentages were calculated from bisulfite sequencing results of multiple independent clones.

Earlier studies have shown that the T-DNA-encoded oncogenes of the virulent *A. tumefaciens* strain C58 are always actively transcribed in crown gall tumors of *A. thaliana* stems [8]. However, transgenes are known to be frequently methylated. Therefore, we analyzed the cytosine methylation pattern of a 5,429 bp T-DNA segment of the pTiC58 plasmid. This segment consists of the coding sequences (CDS) from the oncogenes *IaaH*, *IaaM* and *Ipt* as well as the two intergenic regions between them (IGR1 and IGR2, Figure 1A). Methylated cytosines in this region were determined by bisulfite sequencing of genomic DNA preparations of stem-derived *A. thaliana* crown galls. This analysis revealed that only 0.94% of all cytosines were methylated in the three coding sequences (CDS) of the oncogenes, whereas the two IGRs were completely devoid of methylated cytosines (Figure 1B).

**Figure 2:**
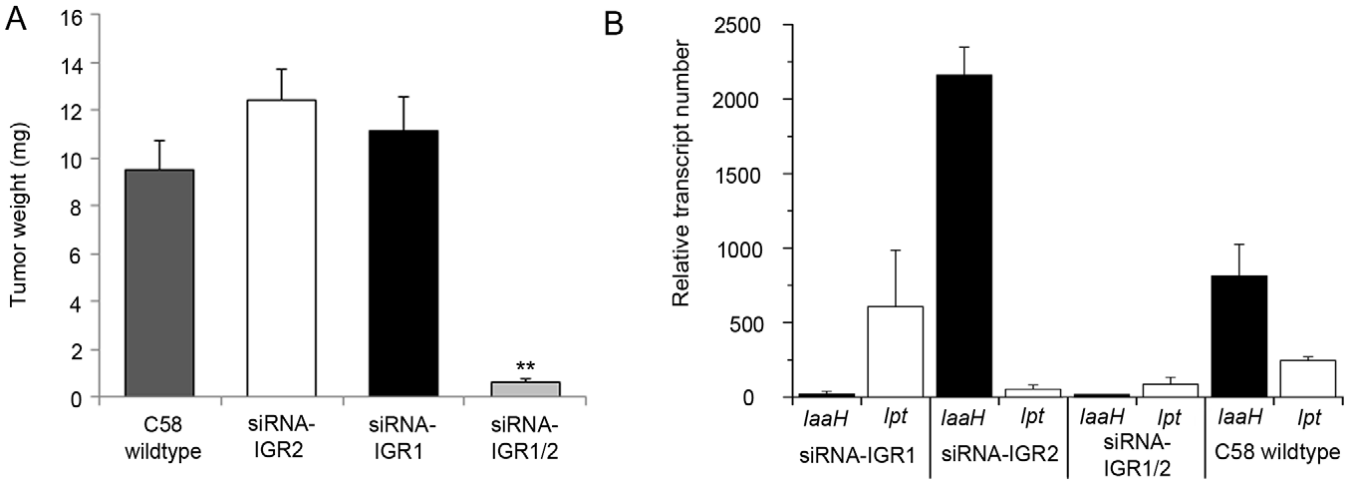
Tumor development and oncogene transcription are inhibited by RNAi–mediated DNA methylation. (A) Crown gall tumor weights were determined four weeks after injection of recombinant virulent *A. tumefaciens* strains of C58 into *A. thaliana* stems (ecotype WS-2). The recombinant strain C58 harbored either an empty pHellsgate12 vector (C58) or a hairpin construct directed against IGR1 (siRNA-IGR1) or IGR2 (siRNA-IGR2) or against both IGRs (siRNA-IGR1/2). Bars represent mean values (± SEM) of at least 38 inoculated plants per *A. tumefaciens* genotype. Statistical analysis was performed using student's t-test: p-value<0.01 (**). (B) Relative transcript numbers of the oncogenes of strain C58 in *A. thaliana* stem tissue six days after inoculation with the *A. tumefaciens* genotypes used for the crown gall tumor growth assay in (A). Relative transcript numbers were quantified by real time qRT-PCR and normalized to 10.000 molecules of *ACTIN2/8*. Bars represent mean values (± SD) of three independent experiments.

The extremely low degree of T-DNA methylation in crown gall cells suggests that this is a prerequisite to maintain the expression levels of oncogenes required for tumor formation. This hypothesis was tested by the induction of oncogene promoter methylation, making use of the endogenous siRNA-directed plant methylation pathway. Plasmids containing a hairpin construct directed against the IGRs upstream of the oncogene CDSs, each fused to the CaMV35S promoter (Figure S1), were transferred into the crown gall genome by using the virulent *A. tumefaciens* strain C58. Development of crown gall tumors was strongly impaired (Figure 2A) when *A. thaliana* was inoculated with strain C58 that contained hairpin sequences directed against both IGRs (siRNA-IGR1/2, Figure S1A). In contrast, no growth inhibition occurred on plants inoculated with strain C58 if only one IGR was addressed by a hairpin construct (Figure S1B). Bisulfite sequencing analysis of both IGRs revealed that the IGR1/2 hairpin induced methylation of cytosines in all three sequence contexts upstream of the *Ipt* and *IaaH* CDS (Figure 1C). However, IGR1 was only marginally methylated in contrast to IGR2.

Induction of IGR methylation by hairpin constructs suggests that expression of the oncogenes may be hindered. Quantification of *IaaH* and *Ipt* transcripts by qRT-PCR in *A. thaliana* stems six days after inoculation demonstrated that the transcription of oncogenes was indeed inhibited whenever strain C58 contained a hairpin construct directed against the corresponding IGR (Figure 2B). Transcriptional silencing of *IaaH* was achieved despite a low cytosine methylation level, which suggests that other factors such as siRNA-mediated histone modification may also play role in silencing of this gene [27], [28]. Wildtype *A. tumefaciens* lacking siRNA induced expression of the two oncogene transcripts as expected. Overall, the results demonstrate that the T-DNA sequence is susceptible to DNA methylation and crown gall development can be efficiently prevented by transcriptional silencing of oncogenes.

### The crown gall tumor genome is globally hypermethylated

In order to analyze whether *A. tumefaciens* causes genome-wide DNA methylation changes in the plant genome, we determined the methylation pattern of *A. tumefaciens*-induced *A. thaliana* crown gall tumors. Tumor growth was induced on *A. thaliana* stems, and tumor-free stem material was used as reference tissue. Genomic DNA from each of three independent tumor and stem samples, was randomly fragmented. Thereafter it was subjected to methylcytosine immunoprecipitation (mCIP), resulting in an enrichment of methylated DNA fragments. Each mCIP sample (3× tumor and 3× stem) was separately hybridized to an Affymetrix *A. thaliana* Tiling 1.0R array in parallel with three non-enriched input controls of each tissue type. This tiling array consists of oligonucleotide probes that represent the *A. thaliana* genome with an average resolution of 35 bp. Hybridization signals allowed the detection of genomic regions consisting of at least five genomically adjacent probes that displayed signal intensities above the local background. These regions were assumed to be enriched by the mCIP procedure and therefore considered to contain methylated cytosines.

In total 15,431 distinct genomic regions were methylated in either tissue type. These regions cover 26,287 kb (22.06%) of the *A. thaliana* nuclear genome. In order to verify the reliability of the methylation profile analysis the distribution of methylation signals was examined for both the tumor and stem genome in four categories of annotated loci: Protein coding genes, transposable elements, pseudogenes and non-coding RNAs (ncRNA). For this purpose the proportion (%) of methylated genes out of the total number of genes showing methylation was calculated at 60 positions from 1 kb upstream to 1 kb downstream of genes and plotted along an abstracted model sequence for each gene category. The highest percentages of methylation at all positions were detected in pseudogenes, where they were almost evenly distributed along the entire sequence (Figure S2). In protein coding genes, methylation was especially enriched in the 3′-half of the transcribed region, whereas it decreased towards the transcription start (TSS) and transcription end sites (TES). The methylation pattern was similar for ncRNAs, except that the overall methylated proportion was higher and did not increase in the transcribed region. In accordance with its presumed function in transposon silencing [28], the transcribed regions of transposable elements were highly methylated. Overall, in the genomes of both tissue types the methylation patterns of the mentioned gene categories are well in agreement with those reported in earlier studies [15], [29].

**Figure 3:**
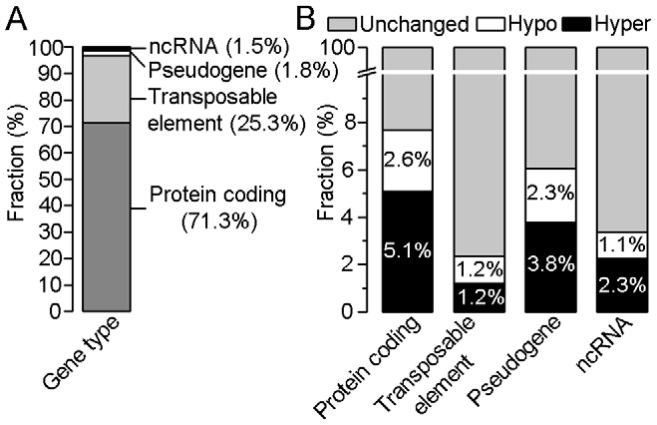
Distribution of differentially methylated regions (DMRs) within four gene types. (A) Percentages of DMRs between crown gall tumors and mock-inoculated stems in four types of annotated loci (protein coding, transposable elements, pseudogene and non-coding (nc)RNA). (B) Percentages of unchanged, hyper- and hypomethylated regions within each of the four gene types. The calculation is based on 2876 DMRs out of which 2052 belong to the group of protein coding genes, 729 to transposable elements, 53 to pseudogenes and 42 to ncRNAs.

In order to identify differences in DNA methylation between crown gall tumor and stem tissue, differentially methylated regions (DMRs) were determined in the four categories (Figure 3; for details see Materials and Methods). This analysis revealed that 2,876 annotated loci differed in methylation levels between crown galls and the tissue from which they originate. The majority of these loci overlapped with regions found to be hypermethylated in crown galls (1,822), whereas 1,100 hypomethylated regions were located in the proximity or inside of annotated loci. The sum of hyper- and hypomethylated regions is higher than that of all affected loci because one locus may contain several DMRs. With respect to the total number of DMRs between crown gall and stem tissue, the majority could be assigned to protein coding genes (71.3%) and transposable elements (25.3%, Figure 3A), which together account for nearly 97% of all DMRs. DMRs were also present in pseudogenes (1.8%) and ncRNAs (1.5%). However, when separately calculating the number of DMRs for each of the four categories of annotated loci, the highest proportion was detected in protein coding genes (7.7%), followed by pseudogenes (6.1%) and ncRNAs (3.4%), whereas only 2.4% of transposable elements were differentially methylated between the two tissue types (Figure 3B). Hypermethylation was more prominent than hypomethylation in protein coding genes (5.1% vs. 1.6%), pseudogenes (3.8% vs. 2.3%) and ncRNAs (2.3% vs. 1.1%), but not in transposable elements (1.2% vs. 1.2%). Taken together, the genome of the crown gall tumor is globally hypermethylated. The changes in the genome wide methylation pattern reflected the increased expression of genes involved in DNA methylation (*MET1*, *DRM2*, *CMT3* and *AGO4*) as well as demethylation (*ROS1/DML1*) in crown galls (Table S1).

In order to verify methylation differences by an independent method, one DMR was randomly chosen from each of the five *A. thaliana* chromosomes and analyzed by bisulfite sequencing. The directions of methylation changes at the tested loci (hypermethylation: At5g58370, At4g12460 and hypomethylation: At3g19250, At1g20850) were in agreement with the results achieved by tiling array analysis, except for the locus At2g16595 from chromosome 2 (Figure S3). This locus was enriched in immunoprecipitated crown gall DNA, which indicates an increase in methylation. However, bisulfite sequencing revealed that only CG and CHH methylation was increased at this locus, but this was accompanied by a decrease in CHG methylation.

Changes in the methylation pattern may be a result of altered ploidy levels which frequently occur in *A. thaliana*. Therefore, the (endo)ploidy levels of *A. thaliana* crown gall cells as well as tumor-free stem cells were determined by flow-cytometry (Figure S4). In both cases the first DNA peak (2C) was found at similar positions, excluding a ploidy change towards tetraploidy in the crown gall tumor cells. Neither the histogram nor the cycle value, defined as the mean number of endoreduplication cycles per nucleus [30], indicated an increased endopolyploidization rate in crown gall (0.784) *versus* tumor-free (0.899) tissues. Furthermore, we observed no peak shifts or changes in the peak width on the histograms derived from the crown gall tissue which might have indicated aneuploidy. These data demonstrate that hypermethylation in *A. thaliana* crown gall tumors is not due to an increased DNA content per nucleus. The significantly decreased 4C/2C ratio in crown galls (1.11) compared to stem tissue (2.32) indicates an increased number of 2C nuclei (Figure S4B) and thus an elevated rate of cell division in crown galls. Whereas in the non-tumor tissue more and more cells switch from the initial mitotic divisions to endoreduplication cycles, tumor cells tend to proliferate mitotically resulting in 2C cells at the end of each cycle.

### Methylation changes occur mainly at non–CG motifs and affect crown gall tumor development

In contrast to the animal genome, a substantial amount of cytosine methylation occurs in non-CG contexts in plants. To identify the sequence motifs which were mostly affected by differential DNA methylation in *A. thaliana* crown gall tumors, all methylated genomic regions were grouped into three classes (hypomethylated, unchanged or hypermethylated). These classes indicate the methylation levels of crown gall DNA compared to control tissue. Pairwise Wilcoxon rank sum tests were conducted to determine whether the frequencies of the three sequence motifs (CG, CHG or CHH) differ between the three classes. Differences in methylation frequency were much less significant for the CG motif (P-value>0.01, Figure S5) than for CHG- and CHH motifs (P-value<0.01). This suggests that methylation changes in the crown gall tumor mainly occurred at CHG and CHH motifs and to a lower extent at CG nucleotides.

**Figure 4:**
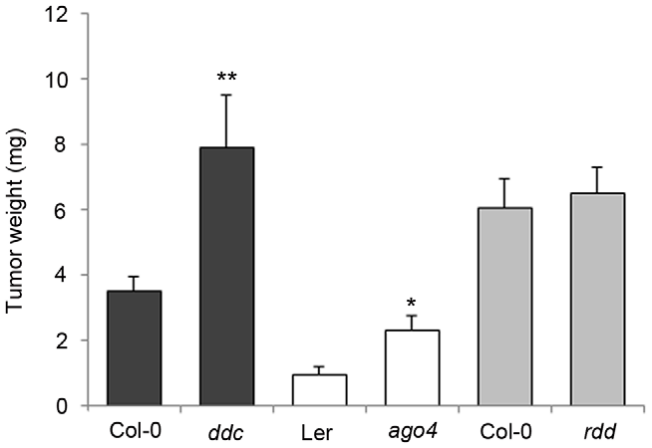
Tumor growth is enhanced in DNA methylation mutants. Tumor weights were determined four weeks after infection of methylation mutants (*ddc* and *ago4*) and the *rdd* demethylation mutants. Plants were inoculated with the virulent *A. tumefaciens* strain C58 at the base of the inflorenscence stem. Error bars represent mean values (± SEM) of at least 45 plants per *A. thaliana* genotype. Statistical analysis was performed using student's t-test: p-value<0.05 (*); p-value<0.01 (**).

The significant changes in the DNA methylation pattern prompted us to test its impact on crown gall development. Several *A. thaliana* mutants with no obvious growth phenotype but with defects in either methylation or demethylation processes were inoculated with the virulent *A. tumefaciens* strain C58. The fresh weight of mature crown galls from both mutant and wildtype plants was compared after 28 days (Figure 4). The *ddc* triple mutant, in which CHG and CHH methylation are strongly impaired, displayed significantly enhanced crown gall growth. A similar difference in tumor growth was found between wildtype plants and the *ago4* mutant, which is impaired in RNA-dependent methylation processes. Note that the differences in tumor weights between *ago4* plants and the *ddc* mutant are likely to be based on their genetic background. Plants in the Ler background of *ago4* are known to develop much smaller crown gall tumors than plants in the Col-0 background of *ddc*. The growth of crown gall tumors was not altered in the *rdd* mutant, which demonstrates that demethylation pathways are not essential for *A. tumefaciens*-induced tumor development. Enhanced growth of crown galls on mutants that are affected in non-CG methylation pathways suggests that hypomethylation at CHG and CHH motifs facilitates plant tumor proliferation. Together with the increased differences in non-CG motif frequency in DMRs, these results provide further evidence for a prominent role of non-CG methylation during crown gall development. Nevertheless, we cannot rule out an involvement of CG methylation, because homozygous *met1*-3 mutants are not suitable for tumor growth assays due to severe developmental abnormalities.

### Distribution of DNA methylation changes and its impact on gene expression

**Figure 5:**
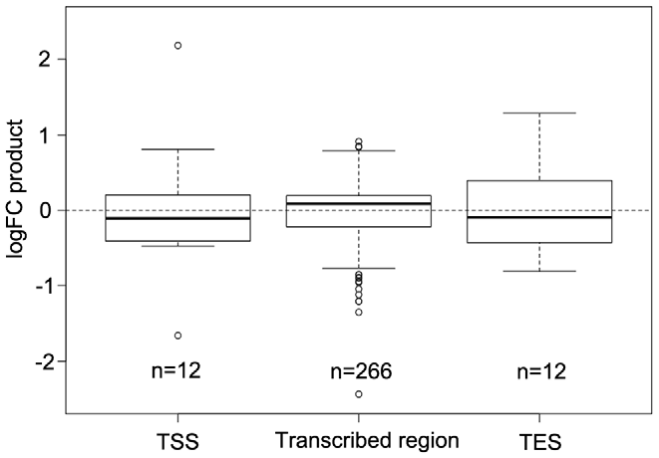
Comparison of differential DNA methylation and differential gene expression. Logarithmic fold changes (logFCs) were determined for differentially methylated regions (DMR) as well as for differentially expressed genes. Methylation logFCs for DMRs and logFCs of differential transcript levels mapping to the transcriptional start site (TSS), the transcribed region and the transcriptional end site (TES) were multiplied and the products displayed as boxplots. Negative logFC products indicate that changes in methylation are associated with gene expression changes in the opposite direction. Positive logFC products imply changes of methylation and gene expression in the same direction.

DNA methylation especially at transcriptional start sites (TSS) and transcriptional end sites (TES) of protein encoding genes and in the transcribed region of transposable elements is known to affect both gene expression and transposon mobility in plants. In order to determine where the changes in DNA methylation preferentially occur, the percentages of hyper- and hypomethylated regions out of all DMRs were calculated for the tumor genome in comparison to the uninfected stem at 60 positions from 1 kb upstream to 1 kb downstream of genes. The distribution of DMRs was plotted along a model sequence for protein coding genes and transposable elements. In the crown gall genome, hypomethylated regions dominated in transcribed regions of transposable elements, while the distal 5′- and 3′-flanking sequences were rather hypermethylated (Figure S6A). Protein coding genes were preferentially hypermethylated in the 3′-half of the transcribed region, whereas both the upstream sequence and the 5′-half of transcribed region were hypomethylated in crown galls compared to stems (Figure S6B). The proportion of hypomethylation in the upstream sequence and around the TSS was relatively high, which may be a mechanism to regulate gene expression in the plant tumor. A comparison of the methylome with transcriptome data from a previous study [8] supported this hypothesis. Methylation changes at the TSS or TES had an inverse effect on gene expression for the majority of genes (negative logFC product). In contrast, differential methylation within the gene body was preferentially associated with gene expression changes in the same direction (positive logFC product; Figure 5).

**Figure 6:**
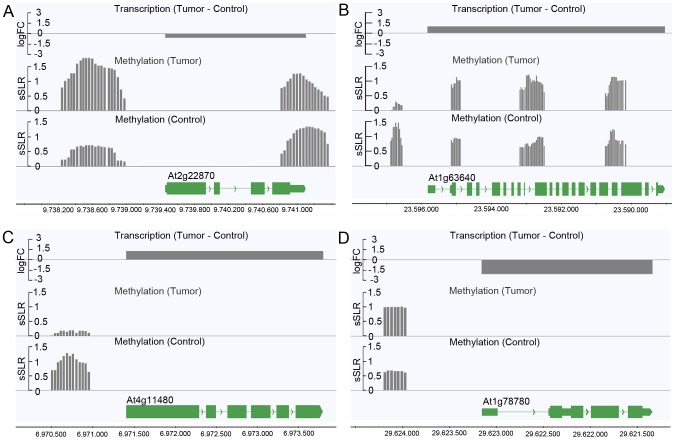
Differences in the degree of methylation in upstream regions correlate with differential transcription. Differential transcription between crown galls and mock-inoculated stem tissue (control) and the DNA methylation status are depicted for selected genes listed in Table S2. The selected genes belong to the following functional categories of Table 1: (A) Development (At2g22870), (B) cell (At1g63640), (C) signaling (At4g11480), and (D) biotic stress (At1g78780). Transcriptional differences were calculated as logarithmic fold changes (logFC, grey bars in the top row). Regions of DNA methylation in tumor and control tissue (grey bars in the 2^nd^ and 3^rd^ row from the top) are shown as smoothed signal log ratios (sSLRs, mCIP versus input). The gene models (green) are displayed according to their corresponding genomic positions at the bottom of each subfigure.

Due to the relationship between the methylation patterns and gene expression levels, a gene ontology analysis was performed to determine which pathways are mostly affected by differential methylation. According to the MapMan software [31], [32] a large number of DMRs that target protein coding genes were enriched in the functional category “development” (FDR≤0.002; Table 1). DMRs were also overrepresented (FDR<0.1) in the categories “cell” (particularly in the subcategory “cell division”), as well as “signaling”, “biotic stress” and “cytochrome P450”. Most of the genes in these categories are involved in processes that are associated with crown gall development; such as transcriptional regulation, cell cycle, chromosome condensation, redox and disease resistance. These differences in methylation correlated with the differences in transcription, as exemplified in Figure 6 for genes involved in embryo development (At2g22870, Figure 6A), microtubule-based movement (At1g63640, Figure 6B), cysteine rich receptor kinase signaling (At4g11480, Figure 6C), and pathogenesis-related protein signaling (At1g78780, Figure 6D). A complete list of all genes affected by differential methylation in the respective pathways is present in Table S2

**Table 1:** Enrichment of protein coding genes with differentially methylated regions (DMRs) in functional categories.

Category name	Gene number with DMRs	Total gene number in category	Fold enrichment	FDR adjusted p-value
development	72	315	2.0	0.001521296
development.unspecified	66	289	1.9	0.002022296
cell	87	458	1.5	0.077132288
cell.division	18	60	2.8	0.082083656
misc.cytochrome P450	24	91	2.3	0.082083656
signalling	120	686	1.4	0.09843198
stress.biotic	73	385	1.5	0.09843198

One-sided Fisher's exact tests were employed to assess the significance of functional categories with DMRs. Listed are the number of genes with DMRs with a false discovery rate (FDR) <0.1. Gene ontology enrichment analysis was performed by applying the pathway analysis program MapMan [31], [32].

### Abscisic acid induces promoter methylation

The significant changes in DNA methylation in crown gall tumors and their role in tumor development piqued our interest regarding the control of physiological processes by DNA methylation. Previously we had shown that the lack of an intact epidermis causes induction of ABA-dependent protection against drought stress in crown galls. Drought stress acclimation is associated with altered transcript levels of many genes involved in ABA-dependent signaling. Acclimation to drought stress is also important for crown gall tumor growth, which has been shown to be impaired in ABA-deficient or -signaling mutants [7]. In order to assess whether ABA influences DNA methylation processes in crown galls, genes known to be strongly transcriptionally repressed by ABA according to the Genevestigator database [33], [34] were selected. In addition to transcriptional regulation by ABA, these genes were known to be significantly downregulated and highly methylated in their promoter sequence in the crown gall tumor. The genes are involved in chloroplast-specific processes, such as cyclic electron flow around photosystem I (*NDF4*), light-dependent transcription of the photosystem II subunit proteins D2 (*SIG5*) and alpha-/beta-hydrolase activity in the chloroplast (*F12A4.4*).

**Figure 7:**
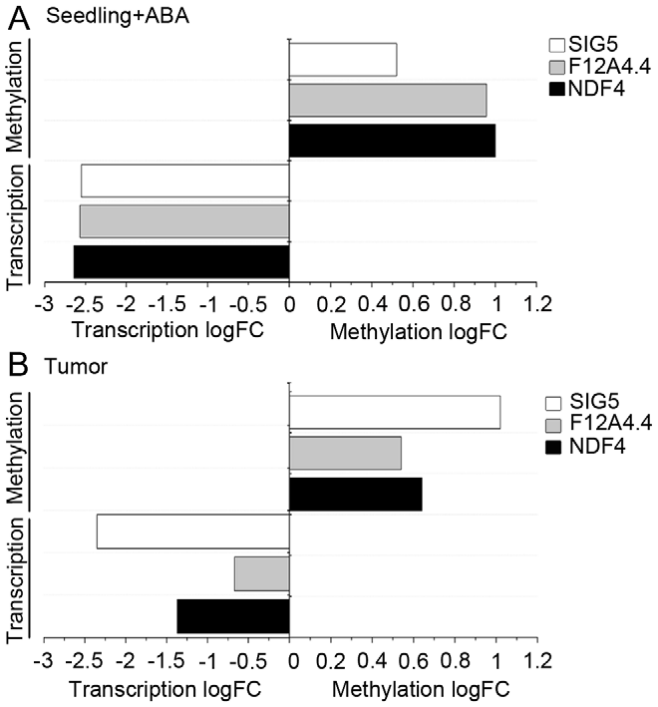
ABA induces methylation and reduces transcript levels. (A) The difference in the levels of transcription (Transcription logFC) and DNA methylation (Methylation logFC) were determined from samples treated with ABA versus samples not treated with ABA for the *A. thaliana* genes *NDF4* (At3g16250), *SIG5* (At5g24120) and *F12A4.4* (At1g35420). Transcript levels were determined by qRT-PCR and methylation profiles of upstream regions by bisulfite sequencing. For these experiments *A. thaliana* seedlings were treated with ABA for two days according to the protocol of Nishimura *et al*. [34]. (B) Changes in methylation and transcription in the crown gall tumor compared to mock-iocculated stems according to methylome (Methylation logFC) and transcriptome data (Transcription logFC) of a previous study [8]. Each data set is based on at least three independent biological replicates and the logarithmic of fold changes (logFC) were calculated from the mean values.

The influence of ABA on promoter methylation of the selected genes was studied in germination experiments with *A. thaliana* seeds, because these experimental conditions were used in the original study from the Genevestigator data set [34]. Methylation patterns of the promoters of the selected genes were analyzed by applying bisulfite sequencing. ABA treatment of .*A. thaliana* seeds provoked increased methylation levels in upstream regions (Figure S7) of *NDF4* (logFC: 1), *SIG5* (logFC: 0.52) and *F12A4.4* (logFC: 0.96) and additionally caused severely reduced transcript levels (Figure 7A). This result reflects the situation observed in *A .thaliana* tumors, where elevated ABA levels and reduced transcription were accompanied by upstream hypermethylation of the tested genes (Figure 7B). Apparently, part of the difference in methylation patterns and transcription between crown galls and the tissue of origin can be ascribed to elevated ABA levels within the tumor.

## Discussion

In recent years, epigenetic processes such as DNA methylation have received increasing attention focused on their function in development, biotic and abiotic stress responses, as well as genome defense [35]–[37]. In our studies we have focused on the impact of DNA methylation on development and physiology of *A. tumefaciens*-induced crown gall tumors. A precondition for crown gall tumor formation is expression of the oncogenes *IaaH*, *IaaM* and *Ipt*, which are encoded on the agrobacterial T-DNA that is integrated into the plant genome. Therefore, it is not surprising that post-transcriptional gene silencing (PTGS) of these genes by means of RNAi caused resistance to crown gall tumorigenesis in previous experiments [38]–[40]. In these studies the *Ipt* and *Iaa* oncogenes were silenced using hairpin constructs directed against their coding sequences. Apart from such PTGS-dependent processes, transcriptional gene silencing can be induced by *de novo* methylation. Previously this epigenetic mechanism has been used to initiate methylation of the agrobacterial nopaline synthase promoter [23]. However, synthesis of siRNA directed against T-DNA-encoded genes like *IaaM* and agropine synthase is specifically inhibited in developing crown gall tumors [14]. This observation is in agreement with our finding that the sequence of the oncogene cluster *IaaH*, *IaaM* and *Ipt* was unmethylated in *A. thaliana* crown galls [23], despite the proposed ancient role of methylation in genome defense [37]. Thus, suppression of siRNA-mediated oncogene silencing as well as methylation-mediated silencing of oncogene promoters may guarantee unimpeded expression of T-DNA oncogenes which is indispensable for tumor growth. The view that promoter methylation induces transcriptional silencing was further supported by introducing siRNAs complementary to oncogene promoter regions. SiRNA-directed methylation of both promoter regions prevented crown gall development, whereas targeting of only one promoter region still allowed proliferation. The latter result indicates that expression of either oncogene is sufficient for tumor growth, which is in line with earlier studies [41], [42]. Transcriptional gene silencing by promoter methylation is thus an effective tool in transgene silencing, as it was previously demonstrated for the nopaline synthase promoter [43]. Induction of siRNA synthesis complementary to oncogene promoters probably outweighs the antisilencing state induced by *A. tumefaciens*. Consequently, it may provide a potent mechanism to suppress tumor development after *A. tumefaciens* infection.

Methylation analysis of *A. thaliana* crown gall tumors revealed that the genome was globally hypermethylated. Hypermethylation may be attributable to increased expression of *DRM2*, *CMT3* and *AGO4* in crown galls, all of which are involved in RNA-directed DNA methylation pathways. Transcription levels of demethylating enzymes probably do not impact the global methylation level since they affect only subsets of genomic loci [26]. In the crown gall genome mainly CHG and CHH motifs were altered, whereas CG methylation was less affected. This suggests that CG methylation does not play an important role in plant tumor development, although *MET1* expression is also increased in crown galls. CG methylation is known to play a major role in generating meiotically stable epialleles due to spontaneous gain or loss of DNA methylation after propagation of *A. thaliana* over several generations. These transgenerational effects are probably a result of epigenetic reprogramming that occurs after fertilization [44], [45]. In contrast, reprogramming of the transformed plant cells begins with the expression of the T-DNA-encoded oncogenes, causing proliferation of the plant tumor. Epigenetic reprogramming during crown gall tumor formation is more likely to be induced by DRM and CMT3 methyltransferases which govern methylation of CHG and CHH sequence motifs [46]. Consistent with this hypothesis, *A. thaliana* mutants (*ddc* and *ago4*), severely defective in non-CG methylation, developed much larger tumors than the wildtype controls. In the *ddc* mutant, for example, CHG and CHH methylation are reduced from 22% in the wildtype to only 1% and 7% of the total methylcytosines, respectively [47]. This indicates that wildtype plants restrict tumor growth by changes in the methylation pattern of cellular genes which are altered in *ddc* and *ago4* mutants. As the integrated oncogene sequences are already unmethylated in wildtype tumors, growth restriction is most likely a result of differential methylation of endogenous plant genes.

In *A. thaliana* crown galls, the majority of DMRs are located in protein coding genes and transposable elements. However, differential methylation within the group of transposable elements is much lower than that of protein coding genes. This is in agreement with a report demonstrating that methylation of transposon sequences is much more stable than that of protein coding genes [48]. The higher stability of transposable element methylation is not surprising considering that loss of methylation of transposable elements has been shown to result in activation of their movement with occasionally mutagenic consequences [49]–[51].

Methylation changes in *A. thaliana* are known to be caused by endoreduplication which results in increased ploidy levels [12]. This is unlikely to happen in *A. thaliana* crown galls as the genome is rather stable in terms of ploidy level alterations. In contrast to the observed global hypermethylation, promoter sequences of protein coding genes in *A. thaliana* tumors were found to be rather hypomethylated and showed an increased level of gene expression. Methylation changes in transcribed regions were associated with transcriptional changes in the same direction. These observations are in accordance with previous studies of DNA methylation in *A. thaliana* [15], [29]. The studies revealed that gene body methylation was preferentially found in highly expressed genes while methylation near the TSS was associated with low gene expression levels. It is widely accepted that DNA methylation at the TSS inhibits transcription by interfering with transcriptional initiation. More controversy surrounds the role of DNA methylation in transcribed regions that may be important in preventing spurious transcription from internal promoters [29] or exon definition [52]. Overall, the results of this study implicate epigenetic processes, among others, as one mechanism to control gene expression in crown galls.

Little is known about plant signals that may affect the DNA methylation patterns and thereby gene expression. A process associated with *A. thaliana* crown gall development is ABA-dependent drought stress acclimation which was shown to be important for wildtype-like crown gall growth [7]. *A. thaliana* mutants in ABA-signaling (*abi1-1*, *abi2-1*, *abi4-1*) and -synthesis (*aba3-1*) display severely reduced crown gall growth. Under drought stress conditions photosynthesis is very much reduced [53]. Accordingly, in crown galls, which undergo increased water loss due to the lack of an intact epidermal layer [54], genes involved in photosynthetic light reactions are significantly downregulated [8]. The idea that biotic and abiotic stresses give rise to an epigenetic modification by ABA signaling has been put forward earlier [36]. For example, the pea genome has been shown to be hypermethylated as a response to water deficit [55]. In addition, ABA has previously been suggested to play a role in DNA methylation in a study from Khraiwesh *et al*. [56], who found that expression of stress-related genes in *Physcomitrella patens* is regulated by ABA in a methylation-dependent manner. In our studies, ABA-mediated methylation of promoters from photosynthesis-related genes caused their transcriptional silencing. The observed methylation and gene expression patterns were similar to those found in *A. thaliana* crown galls accumulating high levels of ABA. Thus, an increase of ABA levels induces promoter methylation and reduces gene expression. These observations suggest that ABA signaling pathways are interconnected with methylation processes in *A. thaliana* crown galls as a response to environmental stress. Unraveling the molecular mechanism underlying ABA-dependent DNA methylation will be an important task for future studies.

The global methylation patterns of mammalian tumors that are induced by integration of viral DNA into chromosomes, such as from Adenovirus type 12 (Ad12), differ from those of most other mammalian tumors. In Ad12-induced tumors, DNA integration into the eukaryotic genome causes *de novo* methylation in *cis* and *trans,* resulting in a hypermethylated genome [10]. Accordingly, hypermethylation in crown galls may be a consequence of the integration of bacterial DNA into the plant genome and the resulting acquisition of constitutive pathways favoring crown gall formation. Alterations in DNA methylation are most likely attributable to the methylation pattern of the transformed plant cells since it has been shown that almost every cell in crown galls expresses the T-DNA-encoded oncogenes and therefore has the potential for proliferation [8].

Until now, infection of *A. thaliana* with *Pseudomonas syringae* is the only plant-pathogen interaction which has been extensively studied with respect to DNA methylation changes [57]. It is accompanied by alterations in the methylation pattern and gene expression, preferentially of defense genes. In contrast to *P. syringae*, infections with virulent *A. tumefaciens* strains induce cell proliferation and crown gall growth. Consequently, the most severe DNA methylation changes in crown gall tumors were detected in genes involved in development, cell division and signaling. Differential methylation of cell division-related genes in *A. thaliana* crown galls is also in line with the observed increased rate of cell division. For example, the gene encoding a kinesin motor protein (AT1G63640) may contribute to cytoskeleton organization during cell division and cell growth as it is both less promoter-methylated and increasingly expressed in the tumor. Methylation seems to be linked to abiotic stress responses. Both the protein kinase CRK32 (At4g11480), which is upregulated in response to abiotic stress [58], and ABA-dependent photosynthesis-related genes (NDF4, SIG5, F12A4.4) are involved in stress-dependent signaling pathways and are differentially methylated in crown galls. The latter genes are severely downregulated in tumors, which are characterized by a heterotrophic metabolism and strong inhibition of photosynthesis genes. Thus, physiological and developmental adaptations during crown gall tumor growth seem to be controlled by epigenetic processes. Furthermore, these results suggest that in accordance with the prevailing response of the host towards a pathogen (development or defense), distinct sets of genes are regulated by DNA methylation in crown gall tumors and in tissues infected with *P. syringae.*

Taken together, this study demonstrates that essential processes during crown gall development are regulated by methylation, which alters the gene expression pattern and controls tumor development. We propose that hypermethylation of the plant tumor genome is a mechanism which restricts tumor growth, for example by affecting genes which are necessary for development and physiological adaptions. Growth restriction allows long-term coexistence of a developing tumor with the host plant and guarantees its survival.

## Materials and Methods

### Plant material

*A. thaliana* plants were cultivated in growth chambers at 22°C during the light and 16°C during the dark period in 12 h intervals. Plants used in this study included the wildtype accessions WS-2, Col-0 and Ler as well as the mutants *ddc* (*drm1–2 drm2–2 cmt3–11,* [59]), *rdd* (*ros1-3; dml2-1; dml3-1,* [26]) and *ago4-1* [27].

### Tumor induction, ABA treatment, and DNA extraction

Tumors were induced by injecting the nopaline-utilizing *A. tumefaciens* strain C58_noc_ (nopaline catabolism; no. 584; Max-Planck-Institute for Plant Breeding Research) into the base of young inflorescence stalks (2 to 5 cm). Tumor tissue was separated from the host inflorescence stalk 28 d after inoculation under a stereo-zoom microscope (Leica MZ6, Leica Microsystems GmbH) using a scalpel. Mock-injected segments of tumor-free inflorescence stalks of the same age were used as reference tissue.

ABA treatment was conducted according to the protocol of Nishimura *et al.* [34]. In brief, *A. thaliana* seeds (ecotype WS-2) were stratified at 4°C for 4 days and were allowed to germinate on 0.8% agar supplemented with full strength Murashige and Skoog salts and 2% sucrose in the presence or absence of 0.5 µM ABA for two days.

Genomic DNA of all plant material was isolated by applying the DNeasy Plant Mini Kit (Qiagen) as outlined in the manufacturer's protocol.

### Immunoprecipitation of methylated DNA fragments

Genomic DNA (1.1 µg) was sonicated with a Bioruptor (Diagenode) until fragments of approximately 600 bp were obtained. The DNA fragments were heated for 10 min at 99°C and immediately cooled on ice for 10 min. One hundred nanograms of genomic DNA fragments were used as input samples for array hybridization. Immunoprecipitation was performed by incubating 1 µg of sonicated DNA with 10 µg of 5-mC monoclonal antibody (Diagenode) in 600 µl IP-Buffer (10 mM Na-Phosphate Buffer, 0.14 M NaCl, 0.05% Triton X-100, pH 7.0) at 4°C for 12 h. Thereafter 100 µl of Dynabeads Protein G were added (Life Technologies), incubated at 4°C for 3 h and washed twice with 600 µl IP-Buffer for 10 min. DNA elution was performed by vortexing the Dynabeads three times in 200 µl TE buffer with increasing SDS-concentrations (0.1%, 0.5% and 1.5%). The DNA was purified by phenol-chloroform extraction and ethanol precipitation.

### Tiling array analysis

Genomic input and mCIP DNA samples were labeled using the GeneChip Mapping 10K Xba kit and hybridized to GeneChip *A. thaliana* Tiling 1.0R arrays (both from Affymetrix) according to the instructions of the manufacturer. Locations of genomic probes were mapped to the TAIR9 version of the *A. thaliana* nuclear genome sequence. Raw array data of input and mCIP pairs were loess normalized. Signal log ratios (SLRs) of mCIP versus input were calculated for crown gall and mock inoculated stem tissue samples and finally quantiles normalized. Probe SLRs from the three biological replicates of each of the two groups were then subjected to a 500 bp sliding window median smoothing in order to create a robust and smoothed SLR (sSLR) for each probe position in each group. An implementation of the CMARRT algorithm [60], [61] was used for detection of genomic regions with consistently increased sSLRs across at least five consecutive probes for each of the two groups. For all regions displaying signal enrichments, log fold changes (logFCs) of crown gall tumor versus non-tumorous samples were calculated based on median region sSLRs. Thereafter, the distribution of logFCs was determined for the regions found to be enriched in the crown gall tumor as well as the tumor-free group (*D_null_*). Tumor-enriched regions of sSLRs with logFCs greater than the 75% quantile of *D_null_* were classified as hypermethylated, whereas those of sSLRs enriched in the tumor-free group with logFCs less than the 25% quantile of *D_null_* were defined as hypomethylated. These hyper- and hypomethylated regions were classified as differentially methylated regions (DMRs). All other regions were considered unchanged. The analyses were performed in R (http://www.r-project.org) along with the packages IRanges, Ringo and Starr (http://www.bioconductor.org).

### Bisulfite sequencing

Bisulfite conversion of methylated cytosine nucleotides was conducted using the Epitect Bisulfite Kit (Qiagen) according to the manufacturer's protocol. A different number of PCR products, depending on the length of the analyzed DNA fragment were generated from the bisulfite-treated DNA, inserted into the pGEM-T easy vector (Promega) and cloned in *E. coli* XL1-Blue MRF′ cells. For each DNA locus multiple independent clones were sequenced, ten clones each for verification of microarrays, ABA-dependent methylation samples and analysis of oncogene methylation in tumors induced by transgenic Agrobacteria, five clones each for oncogene analysis wildtype tumors. Analysis of oncogene methylation of wildtype tumors was performed by analysis of 15 separate fragments covering the sequences of *IaaH*, *IaaM*, and *Ipt*. Primers used for bisulfite sequencing are listed in Table S3.

### Constructs for oncogene silencing

The full lengths of the two IGRs between the coding sequences of *IaaH*, *IaaM* and *Ipt*, comprising 337 bp for IGR1 and 697 bp for IGR2 (Figure 1A) were separately cloned into pHellsgate12 [62] in sense and antisense orientation (Figure S1). This vector expresses the two self-complementary sequences of Ipt IGR and/or IaaH IGR separated by a PDK intron under control of the 35S promoter *in planta*. For generation of the construct with both IGR sequences, the hairpin cassette including 35S promoter, IGR1 hairpin and OCS terminator was PCR-amplified with primers containing *Spe*I adapter sequences (Table S3). Subsequently, this fragment was inserted into the recombinant pHellsgate plasmid already containing the hairpin construct of IGR2 using *Spe*I restriction and ligation. All recombinant pHellsgate12 plasmids were transformed into the virulent *A. tumefaciens* strain C58.

### Flow cytometric measurements

Nuclei of non-tumorous stem and crown gall tumor tissue were isolated one month after mock injection or injection of *A. tumefaciens* strain C58, stained with propidium iodide (PI) as described previously [63] and analyzed using a FACStar^PLUS^ flow cytometer (BD Biosciences) equipped with an INNOVA 90-C argon laser (Coherent). PI fluorescence was excited with 500 mW at 514 nm and measured in the FL1 channel using a 630 nm band-pass filter. Usually 10.000 nuclei per sample were analyzed.

### RNA extraction, reverse transcription (RT), and qRT–PCR

RNA extraction, reverse transcription and qRT-PCR were conducted as described previously [64].

### Data deposition

The data reported in this paper have been deposited in the Gene Expression Omnibus (GEO) database, www.ncbi.nlm.nih.gov/geo (accession no. GSE37680).

## Supporting Information

Figure S1 Maps of recombinant binary pHellsgate12 vectors used for siRNA-mediated transcriptional silencing of oncogenes. (A) The IPT-IAA pHellsgate vector (siRNA-IGR1/2) contains two cassettes in opposite orientation between the right and left T-DNA borders. Both cassettes comprise a CaMV35S promoter, the intergenic regions of IGR1 (337 bp) and IGR*2* (697 bp) each in sense and antisense orientation, separated by two oppositely oriented introns (Pdk, cat) and OCS terminators. (B) The cassette of the Ipt pHellsgate vector (siRNA-IGR2) only contains two copies of the 697 bp IGR2 in sense and antisense orientation. The IAA pHellsgate vector (siRNA-IGR1, not shown) was identical to Ipt pHellsgate except for exchanging IGR2 (691 bp) with the 337 bp IGR1.
(TIF)

Figure S2 Distribution of methylated regions along the sequences of different gene types. The proportion (%) of genes with methylated regions out of the total number of genes showing methylations was plotted against their positions along an abstracted model. Proportions were calculated separately for tumors and mock-inoculated stems from one kilobase upstream to one kilobase downstream and of the transcribed region of four different types of annotated loci: Protein coding genes, pseudogenes, non-coding (nc)RNAs and transposable elements. The transcribed region (hatched) is displayed by relative positions. TSS, transcriptional start site; TES, transcriptional end site.
(TIF)

Figure S3 Verification of mCIP data by bisulfite sequencing analysis of selected genes. Five genes (one per chromosome) were randomly chosen for DNA methylation analysis by bisulfite sequencing. Methylation changes in the tumor are given as log2 fold change from mCIP data (mCIP logFC). Methylation changes by bisulfite sequencing were calculated separately for CG, CHG and CHH motifs as well as all cytosines (C) as differences of percent methylation in crown gall tumors and tumor-free stems from ten individual clones.
(TIF)

Figure S4 Comparison of endopolyploidy levels in crown gall tumor and tumor-free stem tissue. (A) Representative histograms of stem (left) and crown gall tumor tissue (right) from *A. thaliana* (ecotype WS-2). (B) Percentage of individual endopolyploidy levels in stem and tumor tissue, based on five independent measurements.
(TIF)

Figure S5 Sequence motif frequencies of methylated regions in the genome of *A. thaliana* crown gall tumors. The relative number of CG, CHG and CHH motif per nucleotide was calculated for hypo- and hypermethylated as well as unchanged regions. The indicated p-values result from Bonferroni-corrected pairwise Wilcoxon rank tests.
(TIF)

Figure S6 Distribution of hyper- and hypomethylated regions along the sequences of transposable elements and protein coding genes. (A) The percentages of differentially methylated regions between crown gall tumors and tumor-free stems are plotted for hyper- and hypomethylated regions of transposable elements and (B) protein coding genes from one kilobase upstream to one kilobase downstream. Transcribed regions (hatched) are shown by relative positions. TSS, transcriptional start site; TES, transcriptional end site.
(TIF)

Figure S7 Methylation profiles of upstream regions of *A. thaliana* genes in the absence or presence of ABA. The methylation status was determined by bisulfite sequencing and is visualized by pie charts for each position in *NDF4* (At3g16250), *SIG5* (At5g24120) and *F12A4.4* (At1g35420) two days after germination. Percentages of methylated cytosins are shown color coded for the three different sequence motifs (mCG brown, mCHG blue, mCHH red). The change in overall cytosin methylation (mC) was calculated as logarithmic fold changes (logFC) of the methylated proportion of cytosines in the presence (+ABA) versus the absence (−ABA) of ABA. Ten individual clones were sequenced per sample.
(TIF)

Table S1 Differential expression of genes involved in methylation or demethylation in crown gall tumors of *Arabidopsis thaliana.* Fold changes and P-values were calculated from the expression signals of four microarray data sets each of tumor and mock inoculated stem tissue (reference) as previously described [8].
(XLSX)

Table S2 Enrichment of protein coding genes with differentially methylated regions (DMRs) in functional categories according to the pathway analysis program MapMan. One-sided Fisher's exact tests were employed to assess the significance of functional categories affected by differentially methylated genes. The table is sorted according to the column ‘FDR adjusted p-value’. Shown are only categories with a total number of at least 10 genes.
(XLSX)

Table S3 List of primers for the different experiments. Primers are sorted according to the experiments they were designed for.
(XLSX)
