# Supplementary figures and images for: DNA Methylation Mediated Control of Gene Expression Is Critical for Development of Crown Gall Tumors
Source: PLoS Genet. 2013 Feb 7;9(2):e1003267. doi: 10.1371/journal.pgen.1003267 (PMC3567176; doi:10.1371/journal.pgen.1003267)

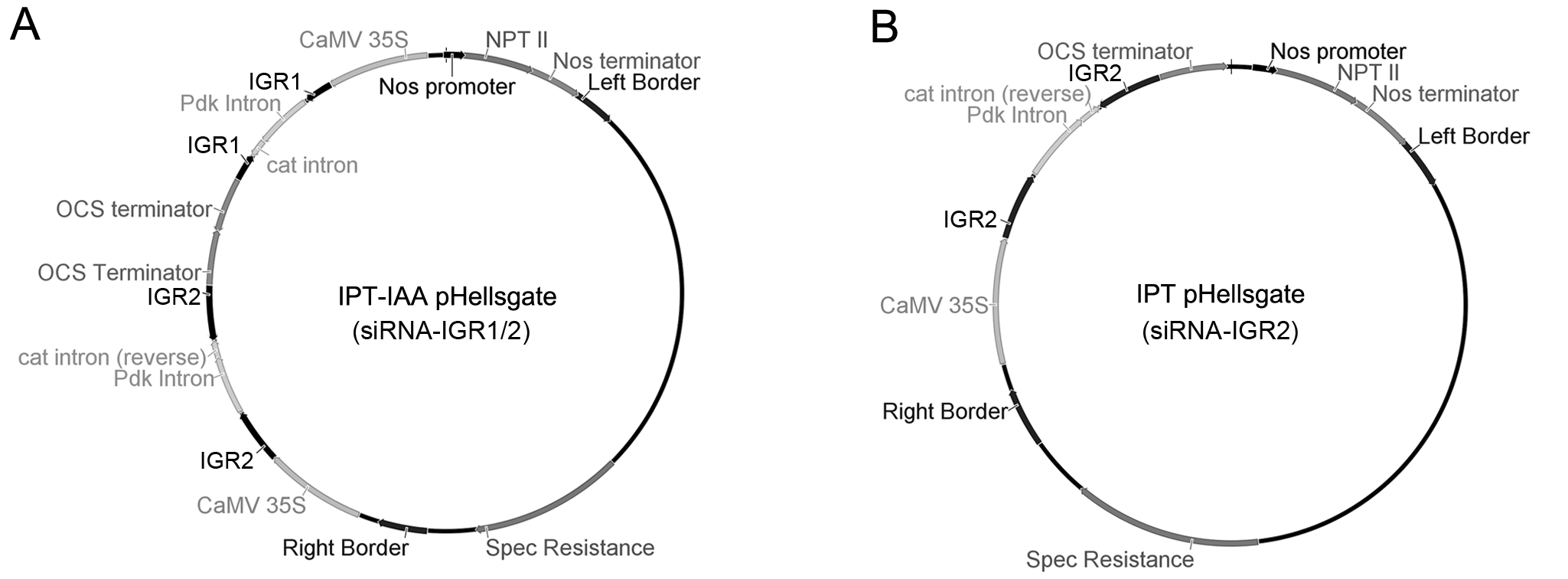

Supplement: Figure S1 — Maps of recombinant binary pHellsgate12 vectors used for siRNA-mediated transcriptional silencing of oncogenes. (A) The IPT-IAA pHellsgate vector (siRNA-IGR1/2) contains two cassettes in opposite orientation between the right and left T-DNA borders. Both cassettes comprise a CaMV35S promoter, the intergenic regions of IGR1 (337 bp) and IGR2 (697 bp) each in sense and antisense orientation, separated by two oppositely oriented introns (Pdk, cat) and OCS terminators. (B) The cassette of the Ipt pHellsgate vector (siRNA-IGR2) only contains two copies of the 697 bp IGR2 in sense and antisense orientation. The IAA pHellsgate vector (siRNA-IGR1, not shown) was identical to Ipt pHellsgate except for exchanging IGR2 (691 bp) with the 337 bp IGR1. (TIF) [file pgen.1003267.s001.tif]

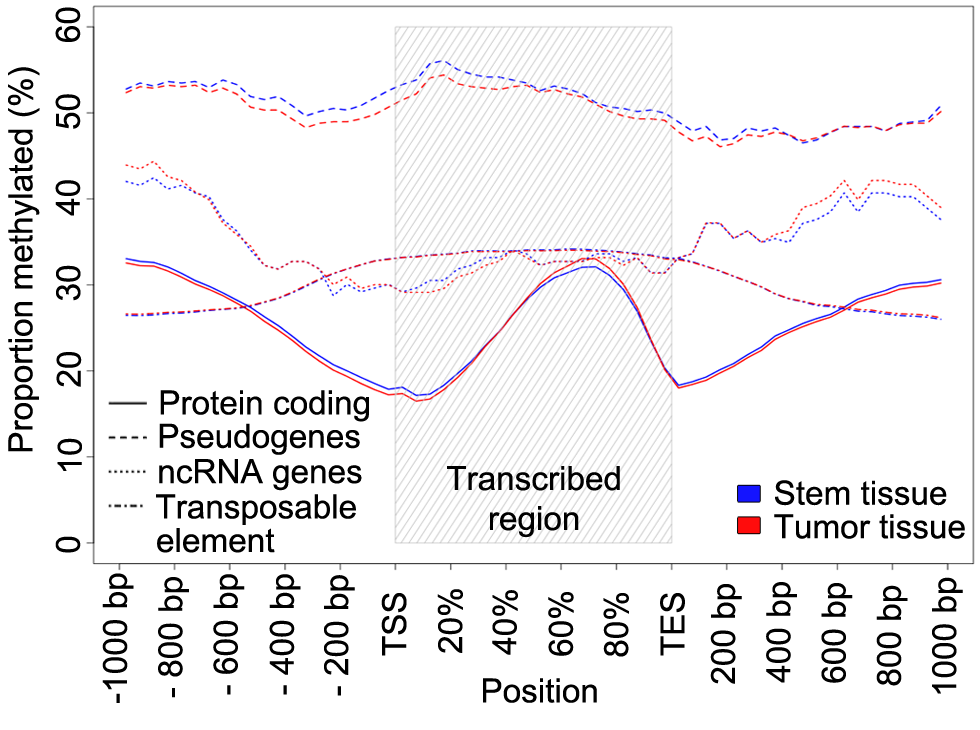

Supplement: Figure S2 — Distribution of methylated regions along the sequences of different gene types. The proportion (%) of genes with methylated regions out of the total number of genes showing methylations was plotted against their positions along an abstracted model. Proportions were calculated separately for tumors and mock-inoculated stems from one kilobase upstream to one kilobase downstream and of the transcribed region of four different types of annotated loci: Protein coding genes, pseudogenes, non-coding (nc)RNAs and transposable elements. The transcribed region (hatched) is displayed by relative positions. TSS, transcriptional start site; TES, transcriptional end site. (TIF) [file pgen.1003267.s002.tif]

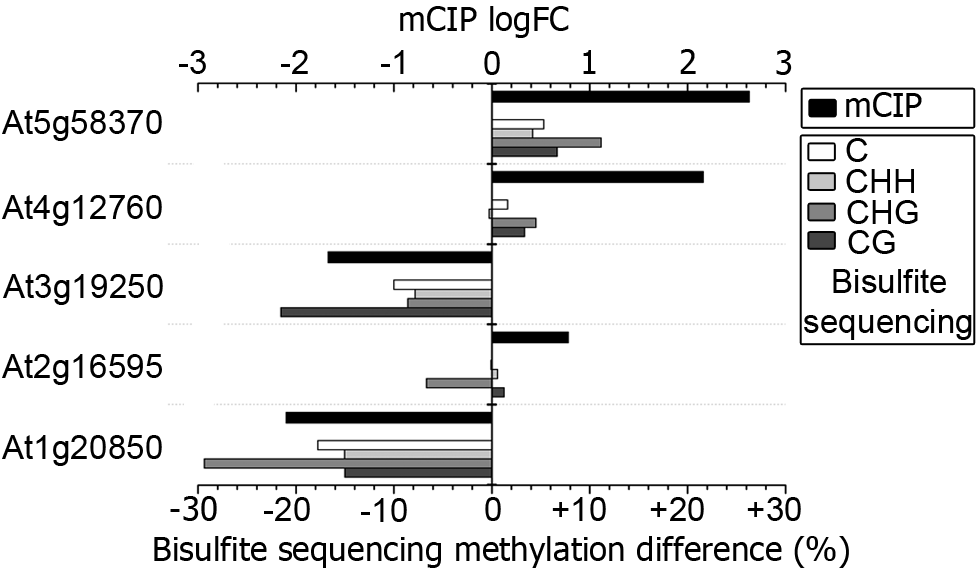

Supplement: Figure S3 — Verification of mCIP data by bisulfite sequencing analysis of selected genes. Five genes (one per chromosome) were randomly chosen for DNA methylation analysis by bisulfite sequencing. Methylation changes in the tumor are given as log2 fold change from mCIP data (mCIP logFC). Methylation changes by bisulfite sequencing were calculated separately for CG, CHG and CHH motifs as well as all cytosines (C) as differences of percent methylation in crown gall tumors and tumor-free stems from ten individual clones. (TIF) [file pgen.1003267.s003.tif]

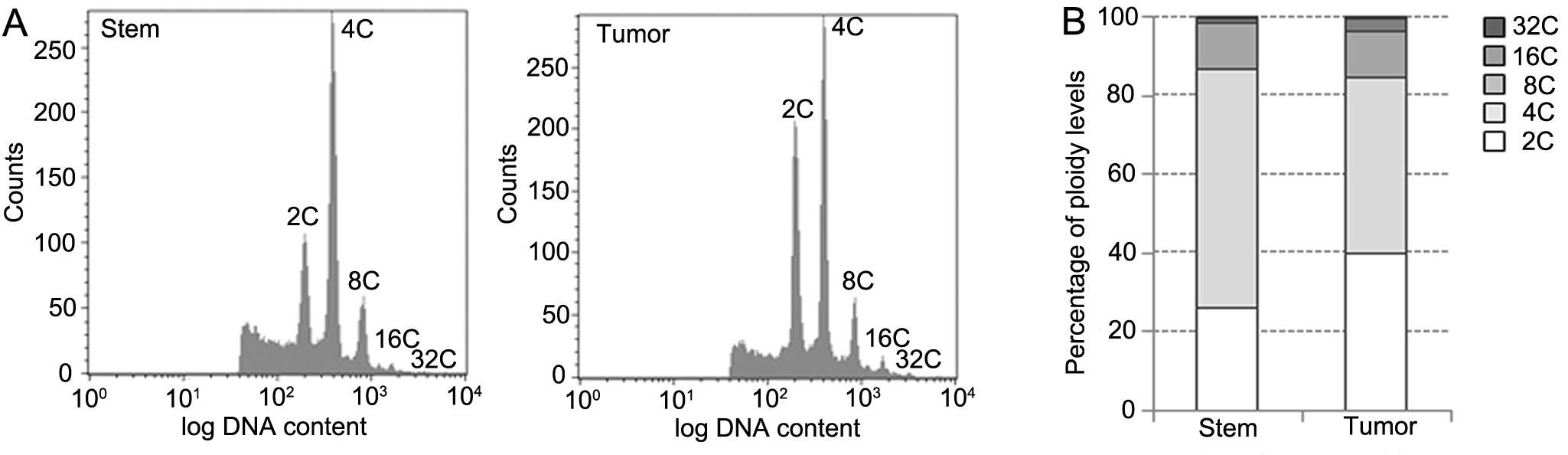

Supplement: Figure S4 — Comparison of endopolyploidy levels in crown gall tumor and tumor-free stem tissue. (A) Representative histograms of stem (left) and crown gall tumor tissue (right) from A. thaliana (ecotype WS-2). (B) Percentage of individual endopolyploidy levels in stem and tumor tissue, based on five independent measurements. (TIF) [file pgen.1003267.s004.tif]

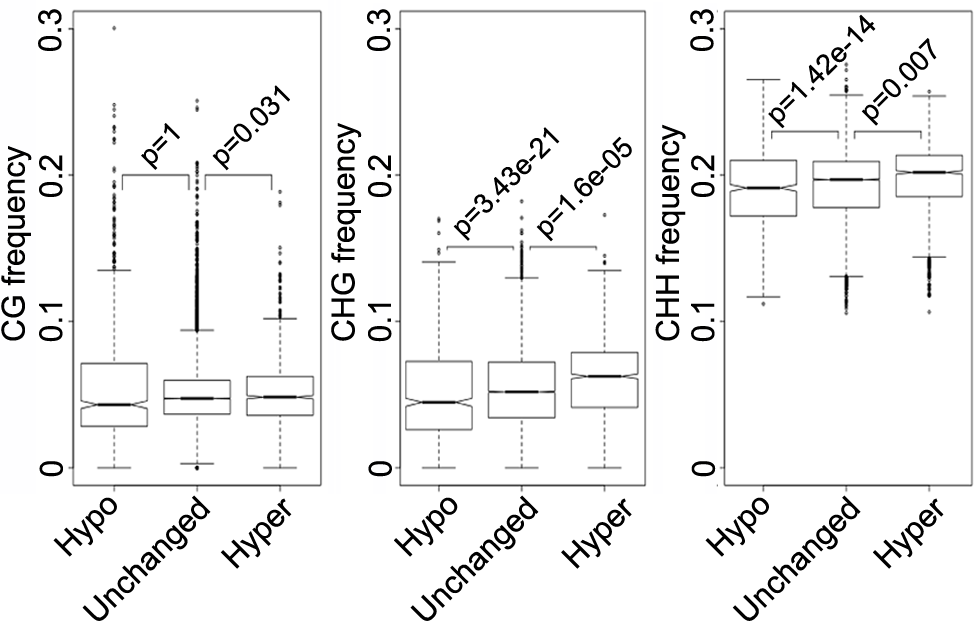

Supplement: Figure S5 — Sequence motif frequencies of methylated regions in the genome of A. thaliana crown gall tumors. The relative number of CG, CHG and CHH motif per nucleotide was calculated for hypo- and hypermethylated as well as unchanged regions. The indicated p-values result from Bonferroni-corrected pairwise Wilcoxon rank tests. (TIF) [file pgen.1003267.s005.tif]

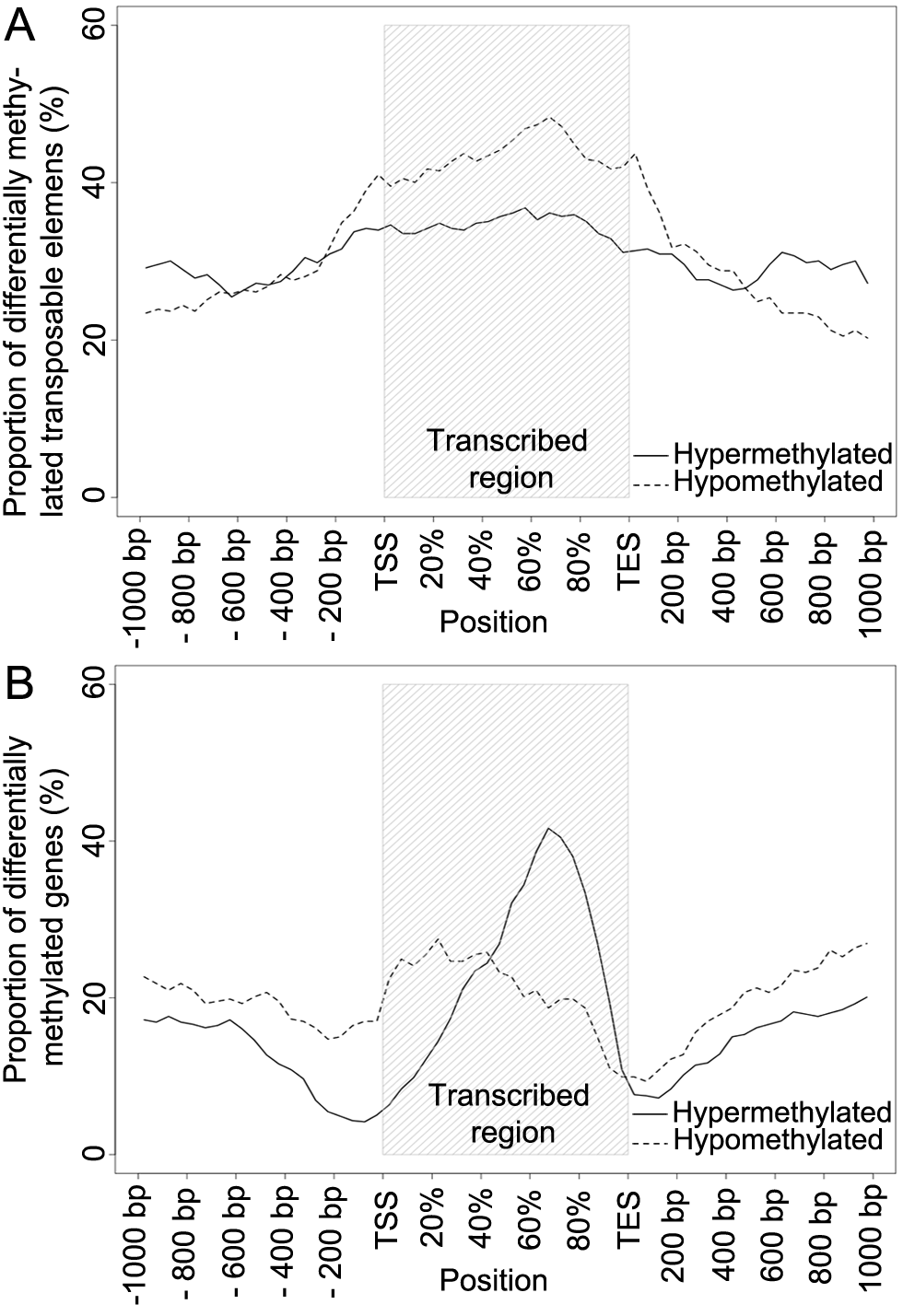

Supplement: Figure S6 — Distribution of hyper- and hypomethylated regions along the sequences of transposable elements and protein coding genes. (A) The percentages of differentially methylated regions between crown gall tumors and tumor-free stems are plotted for hyper- and hypomethylated regions of transposable elements and (B) protein coding genes from one kilobase upstream to one kilobase downstream. Transcribed regions (hatched) are shown by relative positions. TSS, transcriptional start site; TES, transcriptional end site. (TIF) [file pgen.1003267.s006.tif]

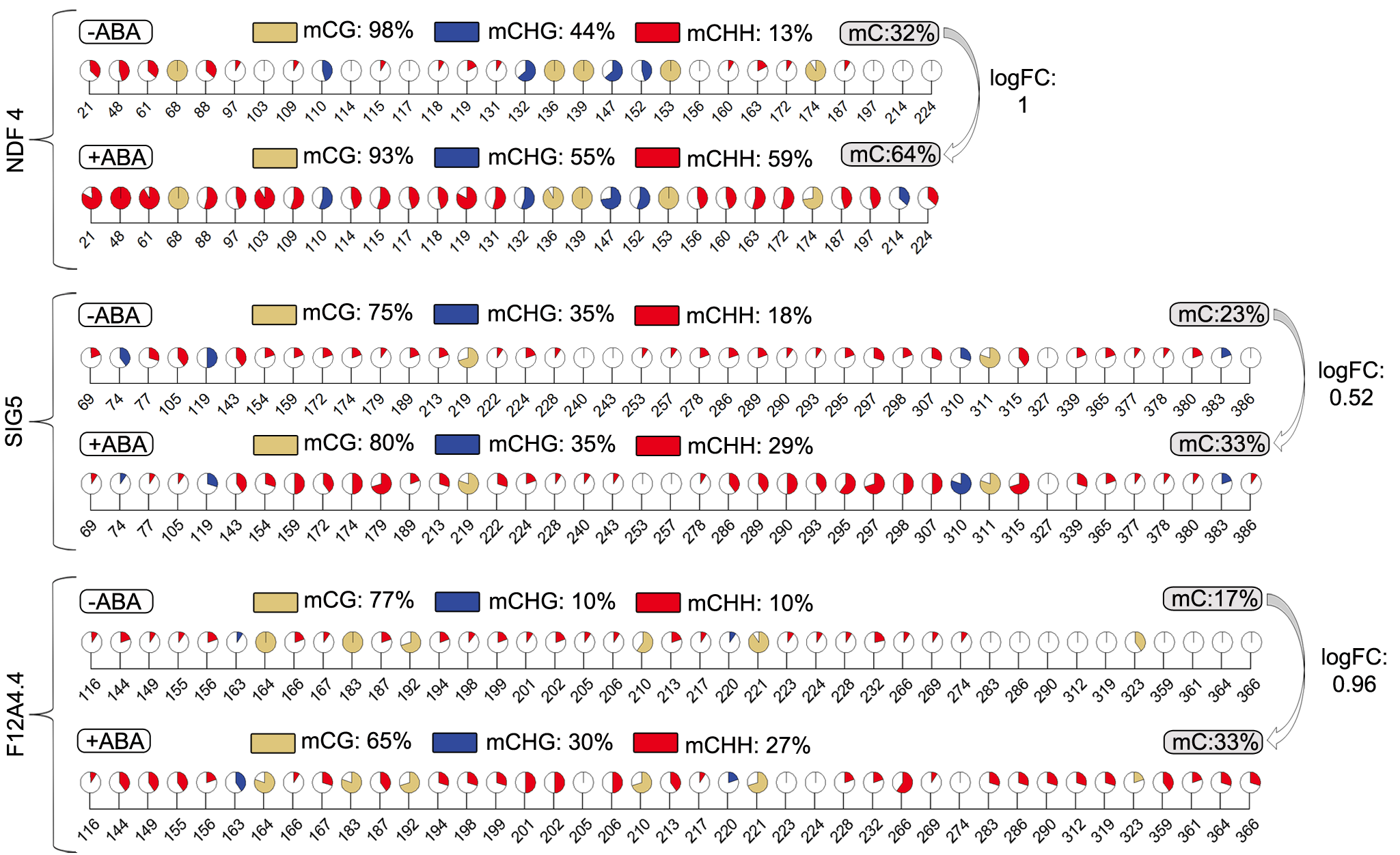

Supplement: Figure S7 — Methylation profiles of upstream regions of A. thaliana genes in the absence or presence of ABA. The methylation status was determined by bisulfite sequencing and is visualized by pie charts for each position in NDF4 (At3g16250), SIG5 (At5g24120) and F12A4.4 (At1g35420) two days after germination. Percentages of methylated cytosins are shown color coded for the three different sequence motifs (mCG brown, mCHG blue, mCHH red). The change in overall cytosin methylation (mC) was calculated as logarithmic fold changes (logFC) of the methylated proportion of cytosines in the presence (+ABA) versus the absence (−ABA) of ABA. Ten individual clones were sequenced per sample. (TIF) [file pgen.1003267.s007.tif]
